# Verbal autopsies of maternal deaths in Koppal, Karnataka: lessons from the grave

**DOI:** 10.1186/1753-6561-6-S1-P2

**Published:** 2012-01-16

**Authors:** Aditi Iyer, Gita Sen, Anuradha Sreevathsa, Vasini Varadan

**Affiliations:** 1 Gender and Health Equity (GHE) Project, Centre for Public Policy, Indian Institute of Management, Bangalore, India

## Introduction

Maternal deaths bear important lessons for death prevention, as they occur mostly due to failures to prevent and respond to obstetric emergencies. These lessons are worth taking. Verbal autopsies can enable such learning and become useful resources for programme managers and policy makers if they identify lapses in death prevention in ways that suggest what corrective actions can be taken.

A review of worldwide literature shows that most verbal autopsy applications to date focus solely on identifying the medical causes of maternal mortality. While such autopsies reveal how the different causes of death are clustered, they do not directly address health system issues. A smaller group of studies examine social causes using the ‘three delays model’ to classify the factors that prevent timely access to medical care in an emergency. However, the model does not directly identify the operational issues that constrain health system functioning nor familial and community-level barriers.

Given this, the Gender and Health Equity (GHE) Project developed a qualitative methodology that departs from existing approaches in significant ways and enables in-depth analysis of the social and medical causes of pregnancy-related deaths.

In this paper, we aim to (1) to describe the methodology developed by the GHE project to analyse and learn from maternal deaths, (2) to identify from the autopsies the types of failures that result in preventable maternal death, as well as the factors that drive them, and (3) to make suggestions for how death prevention measures by public health services can be strengthened.

## Methods

The GHE project tracks on an on-going basis all pregnancy-related deaths that occur in 67 villages of Koppal district, Karnataka. Of these, 23 deaths were investigated using its qualitative methodology through six stages: (1) death notification, (2) data gathering, (3) construction of the narrative and timeline, (4 & 5) analysis of the social and medical causes of death using flow charts and standard guidelines, and (6) identification of corrective actions.

## Results

In resource-poor regions like Koppal, where anaemia and pregnancy-induced hypertension are widely prevalent, the period prior to the emergency is as important for death prevention as the emergency itself. However, in their inability to accurately detect and effectively manage risks, health providers fail the women more critically than do their families.

Demand generation by ASHAs and the availability of the 108 emergency ambulance services result in women going to primary health centres (PHC) or community health centre (CHC) for delivery by choice. But the doctors and staff nurses at these facilities are not always able to triage (i.e., identify emergencies and prioritise treatment/referral for them) or render appropriate care when multiple in-patient admissions take place simultaneously.

Post-partum haemorrhage is the most common cause of maternal death, but this is at least partly due to inappropriate delivery practices by both government and private doctors (e.g., premature application of fundal pressure to hasten delivery, pulling of the cord to hasten placental delivery) and ineffective treatment of the ensuing haemorrhage from inadequate diagnoses of underlying causes.

The three delays model is not always helpful because it links every failed action to one or another delay while leaving unspecified the factors that underpin it. This is not the case. Inappropriate health provider behaviour and treatment in hospitals that stem from a lack of responsiveness or accountability during emergencies do not cause any delay, except in a tautological sense. On the other hand, the inability of government doctors in PHCs and CHCs to identify emergencies and make effective referrals does result in delays.

## Discussion

The capacities of doctors and their staff in PHCs and CHCs to identify and manage pregnancy-related risks and obstetric complications need to be strengthened through practical problem-solving training programmes on a continuing basis.

To enable more accurate detection of risk, ANC check-ups need to become comprehensive assessments that go beyond self-reports by women and/or their families, as there is a tendency among them to normalise risk. Diagnostic methods in use also need to be accurate.

To enable more effective management of risk, communication between health providers and women/their families needs to be strengthened to improve treatment adherence. The monitoring of risk management requires more meaningful follow up than is evident at present.

Finally, the death review process needs to be strengthened through external reviews and the involvement of independent experts.

